# Pro-Inflammatory Cytokines and Antibodies Induce hnRNP A1 Dysfunction in Mouse Primary Cortical Neurons

**DOI:** 10.3390/brainsci11101282

**Published:** 2021-09-28

**Authors:** Muxue Li, Rachel Hamilton, Hannah E. Salapa, Michael C. Levin

**Affiliations:** 1 Department of Anatomy, Physiology, and Pharmacology, University of Saskatchewan, Saskatoon, SK S7N 5E5, Canada; muxue.li@usask.ca (M.L.); rachel.hamilton@usask.ca (R.H.); 2 Office of the Saskatchewan Multiple Sclerosis Clinical Research Chair, University of Saskatchewan, Saskatoon, SK S7N 0M7, Canada; h.salapa@usask.ca; 3 Department of Medicine, Neurology Division, University of Saskatchewan, Saskatoon, SK S7N 0X8, Canada

**Keywords:** RNA-binding protein, multiple sclerosis, stress granules, heterogeneous nuclear ribonucleoprotein A1, cytokines, autoimmunity

## Abstract

Multiple sclerosis (MS) is an inflammatory disease of the central nervous system with a significant neurodegenerative component. Dysfunctional RNA-binding proteins (RBPs) are causally linked to neuronal damage and are a feature of MS, including the mislocalization of the RBP heterogeneous nuclear ribonucleoprotein A1 (A1). Here, we show that primary neurons exposed to pro-inflammatory cytokines and anti-A1 antibodies, both characteristic of an MS autoimmune response, displayed increased A1 mislocalization, stress granule formation, and decreased neurite length, a marker of neurodegeneration. These findings illustrate a significant relationship between secreted immune factors, A1 dysfunction, and neuronal damage in a disease-relevant model system.

## 1. Introduction

Multiple sclerosis (MS) is an autoimmune, neurodegenerative disease of the central nervous system (CNS) characterized by inflammation and demyelination [1,2,3,4]. Axonal and neuronal damage, collectively referred to as neurodegeneration, are prominent features of MS and are believed to underlie disease progression and permanent disability [5,6,7,8,9,10]. Although the exact causes of neurodegeneration in MS remain unknown, there is significant evidence to suggest that the adaptive immune system is involved [11,12,13,14,15,16,17,18,19]. The adaptive immune system is separated into two divisions, the cellular immune response mediated by T-lymphocytes and the humoral response characterized by antibodies. The T-lymphocyte response, particularly T helper cells (Th), has been heavily implicated in MS pathogenesis. Subsets of Th cells, including Th1 and Th17, release pro-inflammatory cytokines and contribute to CNS damage in MS, while anti-inflammatory phenotypes, such as Th2 cells, may play a more protective role during disease [20,21,22,23,24]. Several studies suggest that the immune system may contribute to RNA-binding protein (RBP) dysfunction, which is a feature of neurons from MS cortex and relevant MS models [25,26,27,28,29,30,31].

Dysfunctional RBPs play a role in neurodegeneration in other neurologic diseases and have only recently been implicated in the pathogenesis of MS and its models [26,27,28,30,31,32,33,34,35]. Neuronal RBP dysfunction is characterized by the mislocalization of the RBP from its nuclear homeostatic location to the cytoplasm and the formation of cytoplasmic stress granules (SGs), which can trigger cellular toxicity [36,37,38]. Previously, it has been shown that MS patients make antibodies to the RBP heterogeneous nuclear ribonucleoprotein A1 (A1), a protein overexpressed in neurons [25]. Furthermore, neurons in the brains of MS patients, in contrast to controls, demonstrate increased incidence of dysfunctional RBP biology, including increased nucleocytoplasmic mislocalization of A1 and its colocalization to SGs [27,29].

These data demonstrate that RBP dysfunction, particularly that of A1, is a feature of MS, but what induces RBP dysfunction is unclear. Previous studies in cell lines implicate pro-inflammatory cytokines and anti-A1 antibodies as inducers of RBP dysfunction; however, this has not yet been tested in primary cell culture systems, which are a more biologically relevant model system. Therefore, we hypothesized that the pro-inflammatory cytokines IFNγ and tumor necrosis factor-α (TNFα) and anti-A1 antibodies would induce A1 dysfunction and damage in primary cortical neurons, a physiologically relevant in vitro model system. Here, we demonstrate that both pro-inflammatory cytokines and autoantibodies, which are characteristic of MS, contribute to dysfunctional RBP biology and damage in primary cortical neurons.

## 2. Materials and Methods

### 2.1. Primary Neuron Culture

Animal procedures were performed in accordance with the Animal Research Ethics Board (AREB). Primary neurons were harvested from C57/BL6 female mice (Charles River, Senneville, QC, Canada), aged 3–6 months, according to a published protocol [39]. Briefly, brains were removed from killed mice and placed into Hibernate A medium (Fisher Scientific, Ottawa, ON, Canada) supplemented with B27 and glutamine (HABG medium). The cortex was dissected from the brain before being cut into 0.5 mm sections using a tissue matrix (Harvard Apparatus, Holliston, MA, USA) placed on ice. Tissue was dissociated using papain and manual trituration before being placed onto an OptiPrep (Millipore Sigma, Oakville, ON, Canada) density gradient and centrifuged. The neuronal fractions were collected, centrifuged, and resuspended in Neurobasal A (Gibco) growth medium. Cells were plated onto poly-D-lysine coated coverslips, incubated for 1 h, and washed with HABG medium. Following the final wash, coverslips were transferred to a 12-well plate containing Neurobasal A growth medium and incubated at 37 °C for three days prior to experimentation.

### 2.2. Treatments

Neurons were treated with mouse recombinant IFNγ (2.5 μg/mL; R&D Systems, Burlington, ON, Canada) or TNFα (2.5 μg/mL; R&D Systems, Burlington, ON, Canada) for 24 h. For antibody experiments, IgG isotype control (Millipore Sigma, Oakville, ON, Canada) or anti-A1 (Millipore Sigma, Oakville, ON, Canada) antibodies were conjugated to an AlexaFluor-488 fluorophore with the Lightning-Link Rapid Antibody Labeling Kit (Novus Biologicals, Burlington, ON, Canada) and added at a concentration of 20 μg/mL to neurons for 24 h. Cytokine and antibody concentrations were selected based on previous studies and preliminary experiments to maximize the effect on RBP dysfunction (data not shown).

### 2.3. Immunocytochemistry

Neurons were fixed with 3.7% formaldehyde, permeabilized with 0.3% Triton X-100 in phosphate-buffered saline (PBS) at room temperature (RT), and blocked with 100% SeaBlock (Fisher Scientific, Ottawa, ON, Canada). Neurons were incubated overnight with primary antibody at 4 °C, then with secondary antibody at room temperature, and mounted using ProLong Gold (Fisher Scientific, Ottawa, ON, Canada. The following antibodies were used: rabbit anti-G3BP (abcam, ab214946 and ab217225, Toronto, ON, Canada), chicken anti-beta tubulin III (Aves Labs TUJ, Burlington, ON, Canada), rabbit anti-A1 (abcam ab197854), and donkey anti-chicken DyLight 405 (Jackson Immunoresearch 103-475-155, Burlington, ON, Canada).

### 2.4. Antibody Quantification

Images were acquired at 40× using a Zeiss Axioscope 7 microscope (Carl Zeiss Canada Ltd., Toronto, ON, Canada. Neurons were considered positive if there was fluorescently labelled antibody within cell bodies or processes, which were demarcated with tubulin. At least 100 neurons were analyzed per treatment and each experiment was repeated at least three times.

### 2.5. SG and A1 Mislocalization Quantification

Quantification of SGs and A1 mislocalization was performed in FIJI/ImageJ (NIH). Neurons were positive for SGs if at least three G3BP-positive SGs were present. At least 100 neurons were analyzed per treatment and each experiment was repeated at least three times.

### 2.6. Neurite Length Quantification

The Simple Neurite Tracer plugin in ImageJ was used to manually trace and measure the lengths of neuronal processes, giving a total neurite length sum for each individual neuron. For each treatment group, at least 20 neurons were randomly chosen for neurite length quantification. Experiments were repeated at least three times for a total of at least 60 neurons being analyzed per treatment.

### 2.7. Statistics

Statistical analyses were performed by one-way ANOVA with Tukey’s post hoc tests (*p* < 0.05) (Graphpad Prism Software v. 9, Graphpad, San Diego, CA, USA).

## 3. Results

### 3.1. Pro-Inflammatory Cytokines Induce A1 Mislocalization, SG Formation, and Neuronal Damage

**Figure 1:**
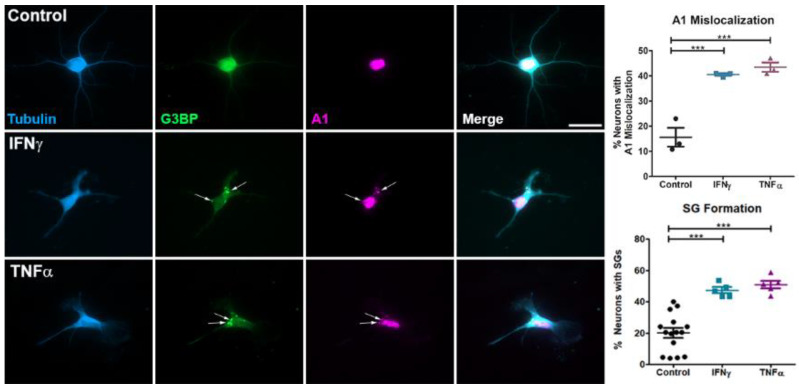
IFNγ and TNFα induce A1 mislocalization and SG formation in neurons. Primary neurons were treated with IFNγ or TNFα and assessed for A1 mislocalization (magenta) and SG formation (G3BP, green). In contrast to control neurons, IFNγ and TNFα-treated neurons displayed significant A1 mislocalization and cytoplasmic SG formation. Mislocalized A1 also colocalized with SGs in both IFNγ and TNFα-treated neurons (arrows). There was no difference in A1 mislocalization or SG formation between the cytokine-treated neurons. One-way ANOVA with Tukey’s post hoc tests with *** *p* < 0.001. Data are plotted as the mean of each replicate +/− SEM. Scale bar 20 μm.

**Figure 2:**
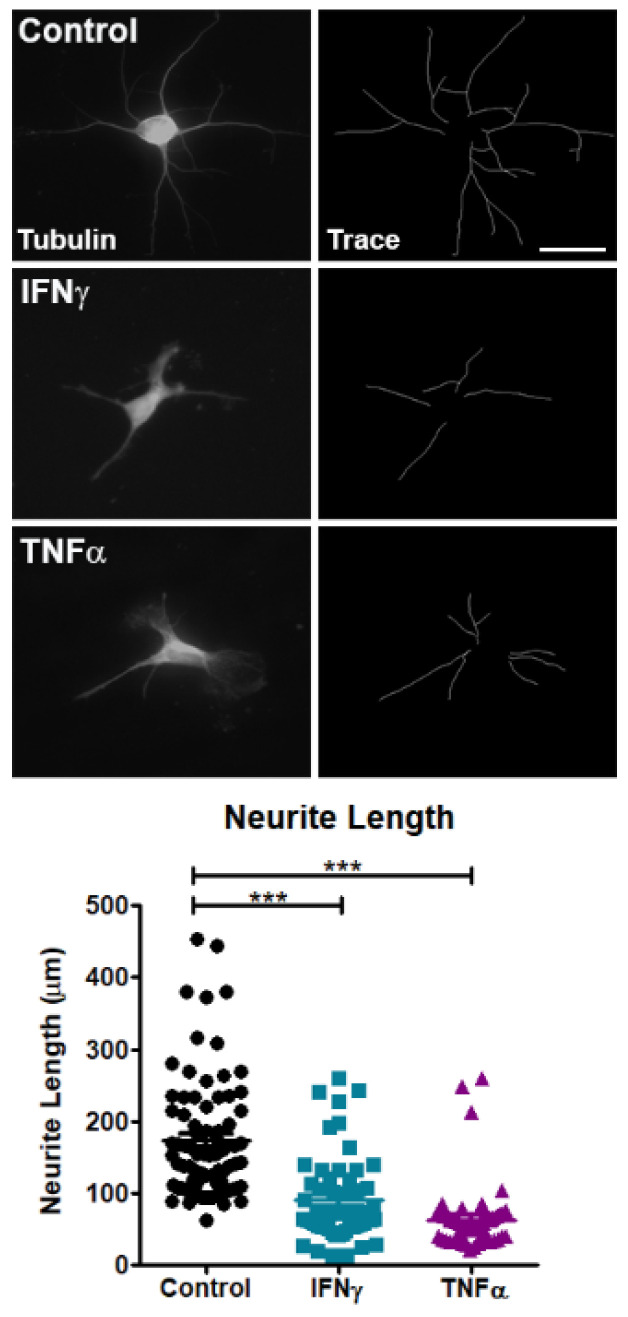
IFNγ and TNFα damage neurites. Primary neurons were treated with IFNγ or TNFα and examined for neurite damage. IFNγ and TNFα led to decreased neurite length as compared to control. One-way ANOVA with Tukey’s post hoc tests with *** *p* < 0.001. Each dot represents an individual neuron with error bars +/− SEM. Scale bar 20 μm.

Previous studies demonstrated that pro-inflammatory cytokines, including IFNγ and TNFα, induce dysfunctional RBP biology in cell lines [27,40]. We hypothesized that the exposure of primary cortical neurons to IFNγ and TNFα would lead to A1 mislocalization and SG formation. In control neurons, A1 was mainly localized to the nucleus (Figure 1). Following exposure to IFNγ and TNFα, significantly more neurons exhibited A1 mislocalization and SG formation as compared to the control (Figure 1). Furthermore, mislocalized A1 colocalized to SGs in pro-inflammatory cytokine-treated neurons (Figure 1). We next examined the effect of pro-inflammatory cytokines on neuronal health using neurite length as an indicator of neuronal damage. Control neurons exhibited extensive processes, whereas IFNγ and TNFα-treated neurons showed significantly decreased neurite length, indicative of neuronal damage (Figure 2).

### 3.2. Anti-A1 Antibodies Enter Neurons and Induce A1 Mislocalization, SG Formation, and Neuronal Damage

**Figure 3:**
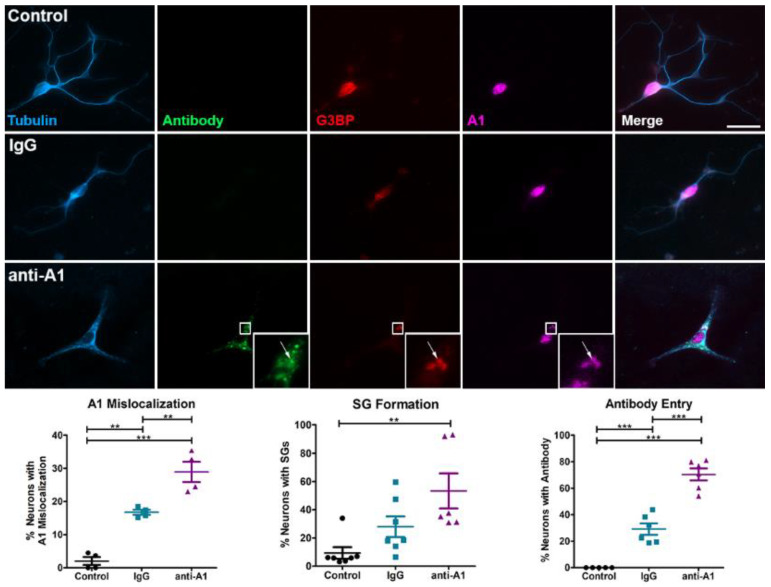
Anti-A1 antibodies enter neurons, leading to A1 mislocalization and SG formation. Primary neurons were treated with anti-A1 antibodies or IgG isotype control antibodies. Anti-A1 antibodies (green) were found to enter neurons at a significantly higher rate than IgG control. Furthermore, neurons treated with anti-A1 antibodies displayed increased A1 mislocalization (magenta) and SG formation (G3BP, red). Arrows indicate colocalization of anti-A1 antibodies, mislocalized A1, and SGs. One-way ANOVA with Tukey’s post hoc tests with ** *p* < 0.01 *** *p* < 0.001. Data are plotted as the mean of each replicate +/− SEM. Scale bar 20 μm.

**Figure 4:**
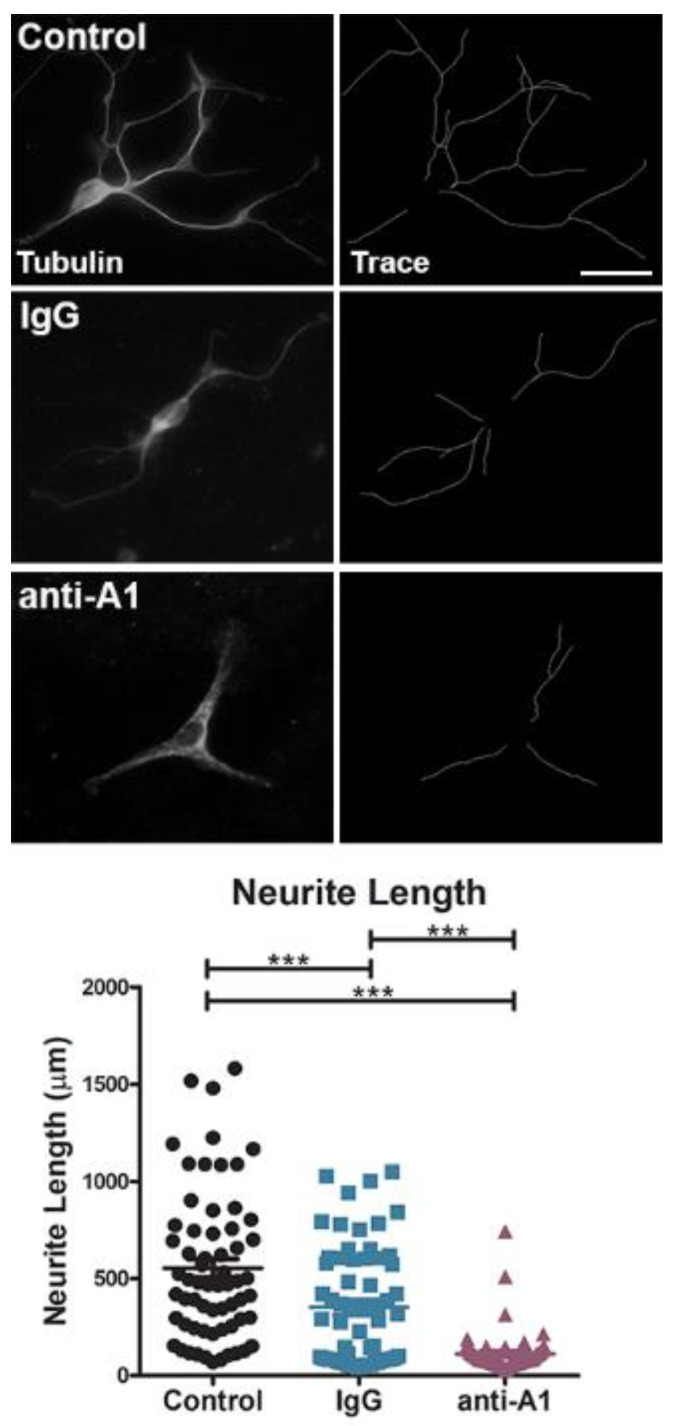
Anti-A1 antibodies damage neurites. Anti-A1 antibodies or IgG isotype control antibodies were added to primary neurons. IgG and anti-A1 antibodies both led to decreased neurite length; however, anti-A1 antibodies augmented neurite damage and led to significantly decreased neurite length as compared to both control and IgG-treated neurons. One-way ANOVA with Tukey’s post hoc tests with *** *p* < 0.001. Each dot represents an individual neuron with error bars +/− SEM. Scale bar 20 μm.

Previous data have demonstrated antibody entry into neurons in several model systems [26,41,42,43]. Therefore, we hypothesized that anti-A1 antibodies might enter primary neurons and affect A1 localization and SG formation. We found that anti-A1 antibodies entered neurons at a significantly higher rate as compared to IgG isotype control antibodies (Figure 3). In addition, neurons exposed to anti-A1 antibodies demonstrated significantly more A1 mislocalization and SG formation as compared to IgG isotype control neurons (Figure 3). Furthermore, anti-A1 antibodies colocalized with SGs and cytoplasmic A1 (Figure 3). Control neurons showed extensive branching and processes, indicative of healthy neurons (Figure 4). Both antibody treatment groups demonstrated significantly decreased neurite length as compared to the control, but anti-A1-treated neurons showed augmented neurite damage as compared to IgG treatment (Figure 4).

## 4. Discussion

Neurodegeneration in MS is the result of a diverse set of cellular mechanisms, including RBP dysfunction. Previous research in cell lines showed a relationship between RBP dysfunction and inflammation [27,40,41,43]. Here, we hypothesized that secreted factors that are characteristic of the immune response in MS, including pro-inflammatory cytokines and anti-A1 antibodies, would induce A1 dysfunction and damage in primary cortical neurons isolated from adult female mice. First, we used pro-inflammatory cytokines that have been demonstrated to play a role in MS pathogenesis, including IFNγ and TNFα [44]. We found that these pro-inflammatory cytokines induce A1 mislocalization and SG formation, two key features of RBP dysfunction, in addition to decreased neurite length, indicative of neuronal damage. Next, anti-A1 antibodies, which MS patients make, were added to primary cortical neurons. In contrast to isotype control antibodies, anti-A1 antibodies entered neurons at a significantly higher rate and induced A1 mislocalization, SG formation, and neurite damage. These data illustrate the relationship between inflammation and A1 dysfunction and neuronal damage in adult primary cortical neurons, a model system that more accurately represents physiological conditions as compared to cell lines.

The effect of inflammation on RBP biology has been previously shown in several cell lines. IFNα, IFNβ, IFNγ, and TNFα have all been shown to induce robust SG responses [27,45,46]. IFNγ and TNFα have been shown to induce RBP mislocalization in cell lines [27,40]. In MS tissues, there is elevated expression of IFNγ and TNFα and their receptors in immune cell infiltrates, neurons, and oligodendrocytes in grey and white matter [44,47]. These data suggest that the presence of pro-inflammatory cytokines within these areas and the cells themselves may contribute to RBP dysfunction and, consequently, neuronal damage. While our data demonstrate that these cytokines individually induce RBP mislocalization, it is not out of the question that these cytokines together may have synergistic effects and amplify RBP dysfunction further. Furthermore, in future studies, it will be important to determine whether this is a characteristic of other pro-inflammatory cytokines (IL-12, IL-18, macrophage migration inhibitor factor) involved in MS pathogenesis [48,49,50,51]. In a similar manner, it is unclear whether this effect is an exclusive feature of pro-inflammatory cytokines or whether anti-inflammatory cytokines (IL-10, IL-13) may also lead to RBP dysfunction [52].

Furthermore, antibodies to RBPs have been implicated in several neurological diseases. Several autoantibodies have been shown to enter neuronal cells in cell lines and neurons in vivo [25,26,41,43]. Furthermore, they have been shown to negatively impact neuronal physiology, morphology, and functioning. Here, we add data to this growing literature implicating pathogenic autoantibodies to RBP targets, where we demonstrate that anti-A1 antibodies enter primary cortical neurons and induce RBP dysfunction and neuronal damage.

While the effects of inflammation on RBP dysfunction have been documented in cell lines, there are insufficient data supporting this relationship in primary neurons. This study is of critical importance as it demonstrates that inflammation induces RBP dysfunction and neuronal damage in primary neurons, a relevant model system. Understanding the mechanisms by which inflammation influences A1 dysfunction and neuronal damage may offer insight into novel targets to correct A1 dysfunction and mitigate neuronal damage in MS and its models.

## Data Availability

Data is contained within the article.
